# p53-Dependent and -Independent Epithelial Integrity: Beyond miRNAs and Metabolic Fluctuations

**DOI:** 10.3390/cancers10060162

**Published:** 2018-05-25

**Authors:** Tsukasa Oikawa, Yutaro Otsuka, Hisataka Sabe

**Affiliations:** Department of Molecular Biology, Graduate School of Medicine, Hokkaido University, North 15, West 7, Kita-ku, Sapporo, Hokkaido 060-8638, Japan; oikawa_tsukasa@med.hokudai.ac.jp (T.O.); h.u.myooh@gmail.com (Y.O.)

**Keywords:** p53, EMT, epithelial integrity, histone modification, miRNAs

## Abstract

In addition to its classical roles as a tumor suppressor, p53 has also been shown to act as a guardian of epithelial integrity by inducing the microRNAs that target transcriptional factors driving epithelial–mesenchymal transition. On the other hand, the ENCODE project demonstrated an enrichment of putative motifs for the binding of p53 in epithelial-specific enhancers, such as *CDH1* (encoding E-cadherin) enhancers although its biological significance remained unknown. Recently, we identified two novel modes of epithelial integrity (i.e., maintenance of *CDH1* expression): one involves the binding of p53 to a *CDH1* enhancer region and the other does not. In the former, the binding of p53 is necessary to maintain permissive histone modifications around the *CDH1* transcription start site, whereas in the latter, p53 does not bind to this region nor affect histone modifications. Furthermore, these mechanisms likely coexisted within the same tissue. Thus, the mechanisms involved in epithelial integrity appear to be much more complex than previously thought. In this review, we describe our findings, which may instigate further experimental scrutiny towards understanding the whole picture of epithelial integrity as well as the related complex asymmetrical functions of p53. Such understanding will be important not only for cancer biology but also for the safety of regenerative medicine.

## 1. Introduction

Epithelial cells may undergo epithelial–mesenchymal transition (EMT) in order to change into highly motile and invasive phenotypes as well as to move out from the original tissue, such as during development, in a manner that is dependent on environmental signals [1,2]. Cancer cells often hijack the EMT process and use this process to further advance the mesenchymal malignancy by enhancing its invasiveness, metastatic ability and therapeutic resistance [3,4]. The recent understanding of the plasticity of cancer stem cell-like cells has demonstrated the complex nature of the mechanisms involved in the EMT process [5] (also see below). Thus, normal epithelial cells might also use yet unforeseen, complicated mechanisms to control EMT.

*TP53* is the most frequently mutated tumor-suppressor gene in human cancers. The functions of p53, the protein product of *TP53*, have been ascribed to its classical role as a transcription cofactor that acts in response to various stress signals to induce cell cycle arrest, cellular senescence and apoptosis [6]. Furthermore, the newly identified functions of p53 include the control of cellular metabolism and antioxidative status [7,8,9,10]. On the other hand, emerging lines of evidence have suggested that p53 might have another function, which is namely to restrict epithelial cell plasticity. For example, p53 can interfere with neural crest delamination, which accompanies EMT [11]. Consistently, mutations in the *TP53* gene (i.e., loss of normal functions of p53) were found to statistically correlate with the generation of cancer stem cell-like cell transcriptional patterns in breast cancers and lung cancers [12]. Interestingly, this generation of cancer stem cell-like properties by the loss of normal-p53 is often coupled with the EMT of cancer cells [13]. p53 can inhibit the generation of induced pluripotent stem cells (iPSCs) through the direct transactivation of microRNAs (miRNAs), namely miR-34a or miR-145, which downregulate pluripotency factors [14,15,16]. In this regard, it has been shown that p53 induces the expression of miRNAs that target mRNAs encoding transcription factors driving EMT (EMT-TFs), such as *ZEB1*, *SNAI1*, *SLUG* (*SNAI2*) and *BMI1* [3,17,18]. This is the prevailing mechanism by which p53 blocks EMT. However, given the cell context-dependent functions of these EMT-TFs [19,20] and the significant enrichment of the motifs for the binding of p53 in the epithelial-specific enhancers [21,22,23], p53 might have additional mechanisms by which it maintains epithelial integrity. In essence, it might be involved in the maintenance of epithelial gene expression, such as *CDH1*, *GRHL2* and *OVOL2*, via occupying epithelial gene loci (Table 1). On the other hand, it should be noted that some cancer cells resist EMT even in the absence of normal p53 [20,24,25]. Likewise, EMT-TFs are not always induced by the loss of p53 and the p53–miRNAs–EMT-TFs axis does not appear to be a cell-autonomous, linear process in significant populations of cancer cells [20,26] (our unpublished results; also see below).

Recent studies have clarified another potential p53 function, which involves the regulation of the epigenome by controlling DNA methylation, histone methylation/acetylation and non-coding RNAs. DNA methylation statuses in mouse embryonic stem cells (ESCs) were shown to be uncontrollable in the absence of p53, resulting in the generation of intraclonal heterogeneity [28]. Mechanistically, p53 was shown to transcriptionally upregulate DNA demethylases, whereas it downregulates DNA methyltransferases [28]. On the other hand, p53 was also shown to directly interact with the K27-trimethylated histone H3 (H3K27me3) demethylase JMJD3/KDM6B [29]. This interaction is likely involved in the demethylation of H3K27me3 at the binding of p53 regions. Consistently, our recent study [30] identified the existence of a safeguard mechanism of p53-mediated demethylation against Enhancer of Zeste Homolog 2 (EZH2)-mediated methylation of H3K27 at the *CDH1* locus, which induces *CDH1* expression in certain cancer cells and organizes them into sheet structures via E-cadherin-mediated cell–cell interactions. Importantly, this function of p53 appeared to be mediated by the direct binding of p53 to an enhancer region of the *CDH1* locus. Therefore, our results may explain the biological significance of the enrichment of the putative motifs for the binding of p53 in the enhancer regions of epithelial genes [21,22,23]. After briefly summarizing recent information on p53 function in epithelial integrity, we aim to discuss the possibility of multi-layered epithelial integrity in terms of its origins and biological significance.

## 2. p53-Mediated Epithelial Integrity via miRNAs and EMT-TFs

p53 functions to restrain epithelial cell plasticity, which partly occurs by negatively regulating factors that initiate and maintain the EMT program. For instance, p53 upregulates MDM2 and forms a complex of p53–MDM2–SLUG to facilitate SLUG degradation, which leads to enhanced E-cadherin expression [31]. In addition, p53 inhibits SNAI1 activity via inducing miR-34, which targets *SNAI1* mRNA by binding to its 3′ untranslated regions (UTRs) [32]. p53 also induces miR-200c to target *ZEB1* and *BMI1* mRNAs by binding to their 3′ UTRs, which inhibits the post-transcriptional processes of these mRNAs [17,18]. *BMI1* encodes a subunit of polycomb repressive complex (PRC) 1, which maintains stem cell functions [33,34] and promotes EMT [35]. A possible direct molecular link between the stem cell properties and EMT was reported by using mammary epithelial cells [13]. Consistently, some breast cancer cells and lung cancer cells lacking normal p53 were shown to exhibit stem cell-like transcriptional patterns [12]. Therefore, the p53–miRNAs–EMT-TFs axis has constituted a prevailing paradigm that explains p53-dependent epithelial integrity [19]. However, it is important to consider that not all epithelial cells undergo EMT in the absence of p53 [24,25]; p53 is also expressed in mesenchymal cells; and p53 limits the reprogramming of fibroblasts to iPSCs by inhibiting mesenchymal–epithelial transition [36]. Therefore, considering this information, p53 appears to have additional mechanisms other than via miRNAs and EMT-TFs for the maintenance of epithelial integrity.

## 3. p53-Mediated Epithelial Integrity via Epigenetic Control

The chromatin structure is dynamically remodeled to control gene expression. Such epigenetic regulation of gene expression can be achieved through the coordinated actions of TFs and DNA/histone modifications. However, such epigenetic modifications in many cases have to be moldable and plastic in order to be delicately balanced between a state strictly maintaining integrity and a state that enables differentiation. Intriguingly, cellular metabolism is critically involved in such processes of epigenetic regulation [37]. For example, the cellular levels of acetyl-CoA, which is the sole donor of an acetyl moiety for protein acetylation, have been demonstrated to affect the degree of histone acetylation and hence, gene expression in mammalian cells [38]. It has been shown that proto-oncogenes, such as Myc and Akt, may promote acetyl-CoA production apart from their classical roles of inducing growth-promoting TFs, thus facilitating histone acetylation to favor cell growth and/or proliferation, which cooperates well with growth-promoting TFs [39,40,41]. Consistently, human embryonic stem cells produce high levels of acetyl-CoA through glycolysis and this production rapidly decreases upon the induction of differentiation [42].

Moreover, p53 has been shown to promote DNA methylation to maintain the silence of some repetitive DNA elements in the mouse genome [43]. Mechanistically, DNA methyltransferases are recruited to the nucleotide regions within these elements where the binding of p53 occurs [43]. On the other hand, a recent study in mouse ESCs demonstrated that p53 transcriptionally represses the de novo DNA methyltransferases *Dnmt3a* and *Dnmt3b*, while upregulating the DNA demethylases *Tet1* and *Tet2* [28]. Consequently, p53 acts to maintain DNA methylation homeostasis as ESCs from p53^−/−^ mice demonstrated both increased DNA methylation and enhanced intraclonal heterogeneity, which might reduce the pluripotency of the ESCs [28].

p53 was also shown to directly interact with the H3K27me3 demethylase JMJD3/KDM6B [29]. Both p53 and JMJD3/KDM6B are upregulated after DNA damage, which subsequently become colocalized at the regulatory elements of well-characterized p53-target genes, such as *CDKN1A* and *MDM2* [29]. Consequently, p53 may be able to decrease H3K27me3 around the p53-target genes, which has been observed during human ESC differentiation [44].

Moreover, p53 may also regulate gene expression at a long distance by inducing enhancer RNAs [45] and large intergenic noncoding RNAs (lincRNAs) [46]. Of note, *Neat1* was recently identified as a p53-induced lincRNA and its deficiency was shown to cause malignancy of pancreatic ductal adenocarcinoma (PDAC) through the global changes in gene expression [47].

## 4. Enrichment of p53-Binding Motifs at Epithelial-Specific Enhancers

The enhancer regions of the *CDH1* locus contain 6 putative sites for the binding of p53 [21,22,23] and this enrichment is comparable to that found at the promoter regions of typical p53-target genes, such as *CDKN1A* (Table 1). On the other hand, it is noteworthy that although the *CDH2* locus encoding mesenchymal-specific N-cadherin also contains 8 putative sites for the binding of p53, none of them are located within the enhancer regions (Table 1). As mentioned above, the functions of p53 are performed by the binding of p53 to nucleotides to activate transcription or by the p53-mediated recruitment of enzymes, such as histone modifiers, chromatin remodelers and RNA polymerase II, to the nucleotide sites that p53 binds to [48]. However, these previously known mechanisms of p53 do not explain why the putative binding motifs of p53 are enriched within epithelial-specific enhancers.

**Figure 1:**
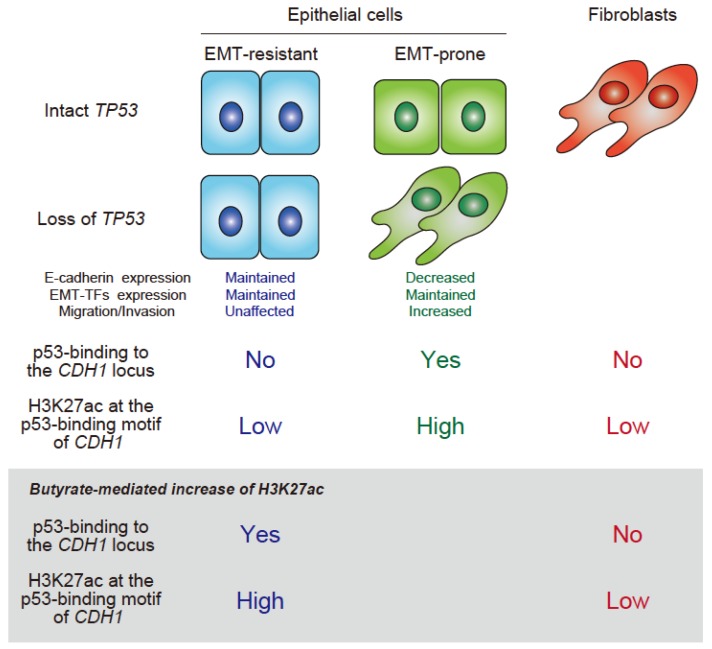
Illustration of the two novel modes of epithelial integrity, which do or do not involve the binding of p53 to the *CDH1* locus. It is important to note that EMT can be induced even in some EMT-resistant cells, such as MCF7 cells in an inflammatory milieu [52].

We recently found that p53 binds to the nucleotides of the *CDH1* enhancer of some epithelial cells, with this binding being necessary to maintain *CDH1* expression [30]. Consequently, in these cells, the loss of p53 causes a loss of *CDH1* expression and CRISPR-Cas9-mediated deletion of the region that p53 binds to abrogates *CDH1* expression. Mechanistically, we showed that the binding of p53 is necessary to maintain high levels of histone H3K27 acetylation (H3K27ac) of the *CDH1* locus, in which p53 appears to antagonize EZH2 activity, a catalytic subunit of PRC2, which otherwise catalyzes the trimethylation of H3K27 (H3K27me3). On the other hand, it is well known that not all epithelial cells need p53 to maintain *CDH1* expression [24,25]. In such cells, we found that p53 does not bind to the *CDH1* enhancer nor needs to antagonize EZH2. Furthermore, we found that the *CDH1* locus was low in H3K27ac compared with p53-dependent cells. Consistently, we later found that high H3K27ac levels lead to the binding of p53. On the other hand, the levels of H3K27ac can fluctuate depending on the cellular acetyl-CoA levels [38]. We demonstrated that although high levels of H3K27ac in *CDH1* can be evoked by butyrate in p53-independent cells, which enables the binding of p53, these epithelial cells still do not rely on p53 to maintain their integrity. Moreover, these p53-dependent and -independent modes of *CDH1* expression were apparently independent of the transcriptional control of EMT-TFs. Our results identified a novel function of p53 that is crucial to maintain epithelial integrity, which operates both in non-transformed and transformed epithelial cells. Therefore, it is likely that the mechanisms involved in epithelial integrity are far more complex than previously thought (Figure 1). Whether this mechanism also operates at the motifs for the binding of p53 found in other epithelial gene enhancers remains to be clarified. We identified *RNF43* [49,50] and *ATP2C2* [50,51] as the candidate epithelial genes that are regulated by the binding of p53. Upon *TP53* silencing, the expression of these genes was reduced and the H3K27me3 deposition around the binding motifs of p53 in their enhancers was increased. The analysis of the Cancer Genome Atlas RNA-Seq datasets of human samples also suggested the existence of the p53–EZH2 antagonism in the regulation of these genes. However, we are yet to perform experiments to obtain convincing evidence that demonstrates the necessity of the binding of p53 to these loci.

Interestingly, the *CDH1* locus in fibroblasts does not show high-H3K27ac nor was affected by butyrate. Thus, these results implied that fibroblasts also have their own mechanism to maintain mesenchymal integrity, which does not enable *CDH1* expression in bona fide mesenchymal cells. Therefore, this poses an outstanding question of whether they occur in vivo and in cells not cultured in vitro for a long time. If this is the case, why epithelial cells need such diversified mechanisms other than the p53-miRNAs axis to warrant their integrity and whether such mechanisms coexist within the same tissues and/or within a single cell are questions that require further research.

## 5. Possible Origins and Biological Significance of the Multiplicity of p53-Mediated Epithelial Integrity

The interactions of genomic DNA and TFs are controlled by a nonlinear process that involves the cooperative actions of histones and metabolites (hence histone modifications) in addition to DNA and TFs. For example, the binding kinetics of SMAD proteins with DNA can be affected by the presence of chromatin modifiers or certain TFs that can physically associate with SMAD. This characteristic of SMAD appears to shape the context-dependent signaling of SMAD [53]. Notably, as described above, the multiple modes of p53-mediated gene expression may generate feedback processes that affect the binding of p53 to the genome, providing an additional layer of nonlinearity. In the steady state of normal epithelial cells as an initial condition, small fluctuations, such as the very small changes in metabolites and histone turnover rates, are thought to be attenuated in order to establish a stable mode of epithelial integrity (e.g., p53-independent). However, such a steady state may become unstable and reach a critical bifurcation point if the cells are exposed to significant perturbations, in which they would transit to another steady state of epithelial integrity (e.g., p53-dependent/EMT-TFs-dependent or p53-dependent/EMT-TFs-independent). Such perturbations may include oncogene expression, which completely alters cellular metabolic states [39,41], or failure in the inheritance of epigenetic memory after cell division [54]. Importantly, the epithelial cell state that undergoes bifurcation can show hysteresis, in which the transition from a steady state to another state is irreversible even in the absence of the original perturbations [55]. An irreversible transition of the steady state is recapitulated in ESCs, in which TFs and extracellular signals cooperatively lead to the fate decision between the epiblast and the primitive endoderm [56]. Theoretical models also suggest that cell fate specification may involve more than 2 stable steady states when the cells respond to external stimuli or internal fluctuations [57,58,59]. Epithelial cells may be intrinsically unstable in their epigenetic landscape as they undergo multiple rounds of cell division and hence, multiple rounds of chromatin inheritance [60,61]. Therefore, we hypothesize that epithelial genes have evolved to harbor multiple motifs for the binding of p53 in their regulatory regions in order to benefit from the binding of p53 through feedback mechanisms from a variety of p53-target genes. Such systems may enable multiple steady states of epithelial gene expression to restrict the easy onset of EMT. Therefore, it will be interesting to investigate the epithelial integrity of primary cultures of normal epithelial cells with or without complete sets of motifs for the binding of p53 at the regulatory regions of epithelial genes, including *CDH1*, in the presence or absence of various stresses and perturbations.

## 6. Perspectives

Apart from studies using ESCs, most studies examining p53 have used cancer cell lines or immortalized epithelial cells or fibroblasts. The p53–miRNAs–EMT-TFs axis was also demonstrated in cell lines with relatively high expression levels of p53 [17,18]. In normal cells, the protein levels of p53 become augmented upon different stresses, such as DNA damage and hyperproliferative signals. Thus, whether the augmented expression of p53 is involved in the p53-dependent epithelial integrity of normal cells remains to be clarified.

The results to date demonstrate that we should go beyond the cultured cells to confirm in vivo the herein described complex mechanisms of epithelial integrity or the complex mechanisms controlling EMT. If these mechanisms do occur in vivo, we thus propose that whether they can be preserved or reproduced during the generation of iPSCs as well as their differentiation into epithelial tissues must be clarified. We identified that the p53-miRNAs axis is not the sole mechanism by which p53 acts to block the cancer mesenchymal programs. Thus, the processes promoting cancer malignancy might also be far more complex than previously thought. In summary, a further understanding of the complex nature of epithelial integrity as well as the processes controlling EMT will advance our understanding on cancer biology and malignancy as well as the safety, usefulness, and limitations of iPSC-based technology.

## Figures and Tables

**Table 1:** The number of motifs for the binding of p53 across the indicated epithelial gene or mesenchymal gene loci (from −10 kb of the transcription start site [TSS] to the end of exons) is listed. The number of motifs for the binding of p53 found in the promoter regions (+/− 5 kb of TSS) of the known p53-target genes is also listed. The motifs for the binding of p53 were identified using the “p53scan” algorithm [27], in which no spacers are allowed between two decameric half-sites.

No. of p53-Binding Motifs across the Gene Locus (No. of Those Proximal to the Cell Type-Specific Enhancer)				No. of p53-Binding Motifs in the Promoter Region	
Epithelial Genes		Mesenchymal Genes		Typical p53-Target Genes	
*CDH1*	8 (**6**)	*CDH2*	8 (**0**)	*CDKN1A*	5
*EPCAM*	3 (**0**)	*VIM*	1 (**0**)	*RRM2B*	2
*TJP3*	1 (**0**)	*ZEB1*	6 (**1**)	*MDM2*	3
*OVOL1*	1 (**1**)	*ZEB2*	6 (**2**)	*GADD45A*	1
*OVOL2*	3 (**2**)	*SNAI1*	0 (**0**)	*TIGAR*	0
*GRHL2*	8 (**4**)	*TWIST1*	2 (**0**)	*BAX*	1
*ESRP1*	4 (**1**)	*TWIST2*	1 (**0**)	*FAS*	1
